# Dispelling Myths and Misunderstandings About Defining and Remediating Dyslexia

**DOI:** 10.1002/dys.70049

**Published:** 2026-09-03

**Authors:** Christy R. Austin, Marissa J. Filderman, Dana Raine, Rose Kjesbo, Courtney D. Boman

**Affiliations:** ^1^ Department of Educational Psychology The University of Utah Salt Lake City Utah USA; ^2^ Department of Special Education The University of Alabama Tuscaloosa Alabama USA; ^3^ Department of Advertising and Public Relations The University of Alabama Tuscaloosa Alabama USA

**Keywords:** dyslexia, dyslexia legislation, pseudoscientific treatments, reading disability, reading intervention

## Abstract

Despite a strong base of scientifically supported research on dyslexia, pervasive misunderstandings continue, particularly in the United States, where state legislation, instructional practices and parent advocacy intersect in ways that influence how dyslexia is identified and treated. Historical misinterpretations about the aetiology and characteristics of dyslexia, evolving definitions of dyslexia and differing ideological beliefs about how to operationalize identifying dyslexia in schools all contribute to uncertainty surrounding what dyslexia is and is not. Misunderstandings have led to a host of fad and pseudoscientific treatments for dyslexia that are unsupported by scientifically based reading research. Misinformation about dyslexia continues to be shared by well‐intentioned, but uninformed, advocates. Disinformation about dyslexia is also shared with education stakeholders for monetary gain. This article draws on scientifically based reading research to (a) illuminate current understandings and misunderstandings about dyslexia, (b) support evidence‐based treatments, (c) dispel fad and pseudoscientific treatments and (d) highlight areas needing additional research.

## Practitioner Points

- Dyslexia reflects word‐reading difficulty along a continuum of severity; students need the same evidence‐based instruction, delivered with greater intensity.
- Structured literacy—explicit, systematic instruction in foundational and language skills—has the strongest evidence for remediating dyslexia.
- Vision‐based treatments, dyslexic fonts and cognitive or ‘brain’ training programs lack evidence for improving reading outcomes.
- Orton–Gillingham's defining multisensory element lacks empirical support; its well‐supported components are shared by other structured literacy approaches.
- Educators and parents should critically evaluate treatment claims, prioritizing instruction grounded in scientifically based reading research.

## 1 Introduction

Many students struggle to acquire proficient reading skills. Current data demonstrates that 67% of United States (U.S.) fourth‐grade students read below the proficient level and 37% read below the basic level (National Center for Education Statistics 2024). While the National Assessment of Education Progress measures comprehension of grade‐level text, few students have isolated difficulties in comprehension (Catts et al. 2006); rather, 85%–95% of students with poor comprehension also demonstrate word‐level reading difficulties (Cirino et al. 2013; Elwér et al. 2015), revealing the challenges schools experience in providing effective word reading instruction (e.g., Walker and Stevens 2017). When word reading difficulties are pervasive in schools, identifying students with dyslexia, whose word reading difficulties fall at the more severe end of the continuum, becomes challenging. Moreover, reading disability is not synonymous with dyslexia. Dyslexia is characterised by primary difficulties at the word‐reading level, with phonological awareness, decoding, encoding and/or oral reading fluency. In contrast, students may demonstrate a reading disability resulting from underlying difficulties with language comprehension, generally referred to as developmental language disorder (DLD), characterised by primary difficulties with language processing, semantics, syntax, comprehension and written expression (Bishop et al. 2017; Snowling 2019). The question remains: Who has dyslexia and how do we provide effective instruction?

In the U.S., dyslexia is currently receiving research and policy attention fueled, in part by parent advocacy groups promoting state‐level dyslexia legislation that extends beyond federal requirements. Frustrated with public schools, parents often turn to advocacy groups they perceive as more responsive and campaign for legislation mandating instruction they believe will best serve their children. As of 2024, 49 U.S. states had passed dyslexia legislation (National Center on Improving Literacy 2024), and we are aware of 37 U.S. states with dyslexia resource guides or handbooks to translate and operationalize the implementation of both federal and state law. However, some policies, legislation and dyslexia guides/handbooks mandate and promote practices lacking scientific support, despite best intentions of advocacy groups (Gearin et al. 2020), resulting in wide use of scientifically unproven treatments for dyslexia (Handler et al. 2011; Kroeze et al. 2016; Ring et al. 2017). The purpose of this article is to (a) illuminate current misunderstandings about dyslexia as a construct, (b) provide support for evidence‐based treatments for dyslexia, (c) dispel fad and pseudoscientific treatments for dyslexia and (d) highlight areas where additional research is needed to inform treatments for dyslexia.

## 2 Understanding Dyslexia

Misunderstandings about dyslexia are pervasive, leading to disagreements about the definition and validity of the construct. We offer a two‐part explanation for misunderstandings about dyslexia: (1) Definitions of dyslexia have evolved over time and continue to vary in emphasis, contributing to confusion about the construct even as they converge on a common description; and (2) Experts disagree ideologically on how to operationalize various definitions of dyslexia.

### 2.1 Evolving Definitions of Dyslexia

Definitions of dyslexia have evolved over several decades, differing in emphasis and terminology even as they have converged on a shared core description. The International Dyslexia Association (IDA) released an updated definition in 2025, the first revision of its widely cited 2002 definition (IDA 2002, 2025). The 2025 definition defines dyslexia as:

a specific learning disability characterised by difficulties in word reading and/or spelling that involve accuracy, speed or both and vary depending on the orthography. These difficulties occur along a continuum of severity and persist even with instruction that is effective for the individual's peers. (IDA 2025)

The IDA's 2025 definition further specifies that dyslexia results from combinations of genetic, neurobiological and environmental influences interacting throughout development, that phonological and morphological processing difficulties are common but not universal, that early oral language weaknesses often foreshadow literacy challenges, and that secondary consequences include reading comprehension problems and reduced reading and writing experience.

The 2025 definition reflects several substantive changes from the 2002 version. The earlier definition described difficulties as ‘often unexpected in relation to other cognitive abilities and the provision of effective classroom instruction’, language that contributed to discrepancy‐based identification and the use of cognitive testing to establish ‘unexpectedness’. The 2025 definition removes unexpectedness language and anchors identification in persistence of reading and spelling difficulties despite effective instruction, aligning more closely with intervention‐responsive identification frameworks (Lyon et al. 2003; Snowling et al. 2020). The 2025 definition also expands aetiology to include genetic, neurobiological and environmental influences, replacing the more circumscribed 2002 framing of phonological deficits as the primary cause. Phonological processing is retained as a common contributor, but morphological processing is added as an underlying difficulty, early oral language weaknesses are named as foreshadowing literacy challenges, and cross‐linguistic variability is acknowledged. The U.S. First Step Act (Public Law 115–391, 2018) defines dyslexia as ‘an unexpected difficulty in reading for an individual who has the intelligence to be a much better reader, most commonly caused by a difficulty in the phonological processing’ (Cassidy 2019), retaining the unexpectedness framing the 2025 IDA definition removes.

Outside the U.S., the United Kingdom's Rose Report (Rose 2009), a government‐commissioned document later adopted by the British Dyslexia Association (BDA 2025), described dyslexia as a learning difficulty primarily affecting accurate and fluent word reading and spelling, characterised by difficulties in phonological awareness, verbal memory, and verbal processing speed. The Rose Report explicitly conceptualised dyslexia as ‘a continuum, not a distinct category’ with ‘no clear cut‐off points’, and emphasised response to ‘well‐founded intervention’ as the primary indicator of severity and persistence. The BDA further noted that some individuals with dyslexia experience visual and auditory processing difficulties and may show patterns of strengths alongside their difficulties (Snowling et al. 2020).

More recently, two complementary Delphi studies have sought to build international consensus. Carroll et al. (2025) convened a multidisciplinary panel of 58 experts who rated statements about dyslexia, accepting those reaching 80% consensus. From 42 accepted statements, the panel concluded that dyslexia is a difficulty in reading and spelling associated with multiple factors, frequently co‐occurs with other developmental disorders, and is marked across ages and language by difficulties in reading fluency and spelling, with phonological processing difficulties the most commonly observed cognitive impairment, though not fully explanatory of individual variability. A companion Delphi study by Holden et al. (2025), focused on assessment and identification practices, similarly converged on intervention‐responsive identification and the need to assess reading fluency and spelling alongside word reading. Together, these studies represent the most recent effort to establish international consensus on dyslexia, and the resulting definition has since been formally adopted by the BDA, superseding the Rose Report definition (BDA 2025).

Definitions of dyslexia in earlier editions of the Diagnostic and Statistical Manual of Mental Disorders (e.g., 4th ed.; DSM‐4; APA 1994) relied on discrepancies between IQ and achievement to identify dyslexia based on the unexpectedness of reading difficulties. However, the DSM‐5 (APA 2013) rejected defining dyslexia based on a discrepancy between IQ and achievement given the lack of evidence for the validity of this approach. Rather, the DSM‐5 required IQ to be within two standard deviations of average to differentiate a learning disability from an intellectual disability, and characterised a reading disability by difficulties in word reading accuracy, fluency and/or comprehension. Difficulties with accurate and efficient word reading are associated with dyslexia, but dyslexia is not a specific category in the DSM‐5.

Although the U.S. IDEA classifies dyslexia as a SLD in basic reading skill, this terminology was not intended to discourage use the term dyslexia. In response to concerns from parents, advocacy groups, and disability organisations, the U.S. Office of Special Education and Rehabilitative Services issued a 2015 guidance letter clarifying that dyslexia is synonymous with a SLD in basic reading skill and encouraged schools not to avoid the term in evaluation, eligibility or IEP documents.

Across definitions and consensus‐building efforts, there is consistent agreement that dyslexia is characterised by difficulties with accurate and fluent word reading and spelling, but differences exist regarding other markers (Miciak and Fletcher 2020; Tonnessen 1997). Some U.S. definitions have historically emphasised the unexpectedness of these difficulties: unexpected underachievement was codified in U.S. federal law through the First Step Act in 2018 (Public Law 115–391; Wagner and Lonigan 2023), while the updated IDA definition, the Rose Report, and the Delphi definition adopted by the BDA do not mention unexpectedness. Critics argue that defining dyslexia in terms of unexpectedness may be inequitable for children from economically disadvantaged backgrounds (Chapman and Tunmer 2019; Odegard et al. 2020), since socioeconomic advantage affords cultural and financial capital to advocate for and obtain a dyslexia diagnosis (Kirby 2020; Nevill et al. 2023), and a child with dyslexia attending a school serving primarily affluent students is more likely to have reading difficulties stand out as unexpected (Odegard et al. 2020). The 2025 IDA definition's shift away from unexpectedness language addresses this critique, although unexpectedness remains embedded in U.S. federal SLD identification statutes.

Definitions also differ in the hypothesised aetiology (Miciak and Fletcher 2020; Tonnessen 1997). Aetiology is absent from the DSM‐5 and IDEA legal definitions, while the IDA's 2025 definition specifies combinations of genetic, neurobiological, and environmental influences interacting throughout development, and the Delphi definition adopted by the BDA similarly states that the nature and developmental trajectory of dyslexia depend on multiple genetic and environmental influences (Carroll et al. 2025). Some definitions present phonological processing deficits as a definitive cause, others state dyslexia *typically* results from phonological deficits, and still others expand the aetiology to include morphological processing or additional cognitive processing deficits such as attention, processing speed and working memory.

Definitions also differ regarding how they address the persistence of reading difficulties (Miciak and Fletcher 2020; Tonnessen 1997). The Rose Report classified dyslexia as a lifelong disorder, and the Delphi consensus similarly recognised that dyslexia persists across the lifespan, although its impact changes with age and context (Carroll et al. 2025). The DSM‐5 operationalises persistence as a diagnostic criterion, requiring that difficulties persist for at least 6 months despite the provision of targeted intervention (APA 2013), whereas the IDEA legal definition does not address persistence directly. Across definitions, persistence serves a common purpose: reading and spelling difficulties that persist despite instruction effective for most students distinguish students with dyslexia from students whose difficulties primarily stem from inadequate instruction. Framing dyslexia as persistent, however, does not imply that intervention is ineffective; rather, students with more severe and persistent difficulties typically require intensive intervention sustained over time. Despite differences in wording and emphasis, these definitions consistently describe dyslexia as a difficulty with accurate and fluent word reading and spelling not attributable to intellectual disability or inadequate instruction.

### 2.2 Differing Ideologies for Operationalising the Definition of Dyslexia

Misunderstandings also stem from differing ideological beliefs about how to operationalize the definition of dyslexia. Researchers conducting scientifically based reading research most commonly use *dyslexia* synonymously with reading disability or SLD (Lopes et al. 2020), specifically as a word‐level reading disability or SLD in basic reading skill (Fletcher et al. 2018). This view positions dyslexia as the lower tail of a normal distribution of reading and spelling abilities (M. Seidenberg 2017; Shaywitz et al. 1992), supports early identification, and recognises dyslexia as difficult to remediate even with generally effective practices, with the cut point for identification viewed as a policy decision (Snowling 2019).

Identifying students with dyslexia and intervening effectively are related but distinct processes. Universal screening can identify students at risk for reading difficulty early, independent of their response to intervention, whereas monitoring students' response to increasingly intensive intervention within multitiered systems of support (MTSS) provides one additional source of identification data while serving the primary purpose of delivering instruction matched to students' needs (Fletcher et al. 2018). Many U.S. schools, however, struggle to implement MTSS effectively (MTSS; Balu et al. 2015), facing challenges with resources, scheduling, and using data to inform instruction. When core classroom instruction is not evidence‐based, distinguishing students with dyslexia from students who have not received adequate instruction becomes difficult (Hanford 2018; M. Seidenberg 2017). Without universal screening and effective MTSS, schools cannot reliably identify where students fall along the continuum of reading difficulty, and those with more severe and persistent reading difficulties, those most consistent with dyslexia, may not receive the intensive intervention they require.

Once students are identified with dyslexia, the interventions provided are often insufficiently differentiated to meet individualised needs, in part because schools struggle to use data to select interventions aligned to specific skill deficits. Not all students with dyslexia exhibit unexpected difficulties at the word reading level, with strong linguistic comprehension; code‐based and meaning‐focused knowledge interact in complex ways to support comprehension (Perfetti and Stafura 2014; Tunmer and Chapman 2012). Many students with dyslexia also exhibit difficulties in linguistic comprehension, including vocabulary and background knowledge between native English speakers and English learners (Catts et al. 2005; Middleton et al. 2024); dual identification with reading comprehension SLD, DLD or speech and language disorder (Snowling 2019); secondary language comprehension difficulties from limited independent reading (Lyon et al. 2003); and cognitive resources taxed by inefficient word reading (e.g., Perfetti 1980, 1985). Interventions failing to address the full spectrum of decoding and language comprehension deficits will be insufficient for students with dyslexia, who demonstrate widely varying needs.

A second ideological position describes dyslexia as a specific type of reading difficulty not identifiable solely on the basis of reading and spelling performance or response to generally effective instruction. Proponents of this position argue for comprehensive psychoeducational evaluation to identify dyslexia, including standardised assessment of reading and spelling abilities alongside measures of cognitive processes such as phonological processing, rapid automatised naming, working memory, and processing speed. The most developed operationalization of this position in the U.S. is the patterns of strengths and weaknesses (PSW) approach to specific learning disability (SLD) identification, which requires evidence of (a) academic weakness in reading or spelling, (b) cognitive processing weakness in an area theoretically linked to the academic weakness and (c) otherwise intact cognitive abilities (Flanagan et al. 2013; Hale and Fiorello 2004; Naglieri and Feifer 2017). Within this framework, cognitive processing deficits are theorised to play a causal role in producing the academic weakness; consequently, identifying the specific cognitive deficits underlying a student's reading difficulty is held to be necessary for both diagnosis and intervention planning. The PSW approach is the most commonly used alternative to response‐to‐intervention models for SLD identification permitted under U.S. federal law (IDEA 2004). However, the PSW approach has been substantively critiqued on empirical and conceptual grounds. Critics note that PSW methods have low diagnostic agreement across approaches (Miciak et al. 2014; Stuebing et al. 2012), that cognitive profiles are unstable across testing occasions and do not reliably predict differential response to intervention (Miciak et al. 2016), and that the cognitive testing required for PSW identification adds substantial cost and time without demonstrated benefit to students' educational outcomes (Fletcher and Miciak 2017).

A third ideological position is more sceptical of the dyslexia construct itself. Stanovich (1991, 1994) argued that distinguishing dyslexic readers from poor readers more broadly lacks robust empirical justification, that IQ‐discrepancy approaches to dyslexia identification have poor predictive validity, and that the cognitive and reading profiles of children with dyslexia largely overlap with those of other poor readers when reading level is controlled. Although Stanovich's position has been refined and contested over subsequent decades, the underlying scepticism has continued in contemporary scholarship. Elliott and Grigorenko (2024), commenting on the IDA definition, argue that the term dyslexia should be reserved for difficulties with accurate and fluent reading, with spelling deficits assessed and identified independently, and that identification should rely on response to high‐quality intervention rather than on cognitive testing. They contend that the position requiring specialist psychometric assessment to differentiate dyslexia from other reading difficulties is unsustainable given the substantial overlap in cognitive profiles between dyslexic and other poor readers. Importantly, this position does not dispute that word reading difficulties fall along a continuum of severity; rather, it questions whether the term dyslexia should be used to demarcate a particular subgroup of poor readers within that continuum, given the difficulty of doing so reliably and the limited implications of the label for intervention. Holders of this sceptical position do not necessarily reject the existence of dyslexia, but they question whether the construct as commonly operationalised adds value beyond accurate description of reading difficulty, and they advocate for pragmatic, intervention‐responsive identification procedures over comprehensive cognitive evaluation.

However, the sceptic position has been substantively contested. Family‐risk and longitudinal studies, discussed in the next section, indicate that children at familial risk for dyslexia exhibit phonological processing deficits before formal reading instruction begins, demonstrating that severe word reading difficulties have identifiable developmental precursors and do not arise solely from instructional experience. Other scholars argue that, while cognitive testing for identification has limited treatment utility, the dyslexia construct itself remains useful for prompting early screening, supporting parental advocacy, and ensuring student access to evidence‐based intervention (Catts et al. 2024; Vaughn et al. 2024). Cognitive‐profile overlap between dyslexic and other poor readers, on this view, does not defeat the construct's validity any more than profile overlap defeats the validity of attention deficit disorders, language disorders or depression—categories which remain valid because they predict developmental trajectory, treatment response and access to appropriate intervention.

Finally, some position and define dyslexia as a different way of thinking affording an individual a range of cognitive strengths and gifts (e.g., Davis 1997; Eide and Eide 2011). Dyslexia has even been considered a ‘desirable difficulty’ (Gladwell 2013); however, there is little scientific support for the ‘dyslexic advantage’. Research is clear: individuals with dyslexia have not been shown to have superior visual–spatial abilities (Gilger et al. 2016) or creative abilities (Erbeli et al. 2022; Majeed et al. 2021).

### 2.3 What Is Dyslexia?

Given various definitions and ideological debates, what exactly is dyslexia? There is agreement that dyslexia is characterised by difficulties with accurate and efficient word reading not attributable to intellectual disability and frequently produces secondary difficulties with reading comprehension and written expression. Its presentation varies across individuals along a continuum of severity shaped by factors including intellect, cognitive processing, comorbid disorders, educational history and linguistic background. Because dyslexia exists along a continuum of severity, effective support also operates along a continuum, not as categorically different types of instruction, but as the same evidence‐based instructional content delivered at increasing levels of intensity to match student need.

### 2.4 What Causes Dyslexia?

Although current definitions converge in their description of dyslexia, consensus regarding its aetiology has been more difficult to achieve. Scientific evidence has nonetheless converged on phonological processing deficits as the most well‐established cause of dyslexia, with morphological processing and additional cognitive processes also contributing to literacy difficulties for many individuals.

#### 2.4.1 The Phonological Deficit

The phonological deficit hypothesis emerged in the 1970s and remains the most empirically supported account of dyslexia (Pennington 2006; Snowling 2019). Phonological processing refers to the detection, manipulation and storage of the sound structure of spoken language, including phonemic awareness, phonological memory and rapid naming. Meta‐analytic evidence demonstrates that phonemic awareness is among the strongest pre‐reading predictors of later word reading (Melby‐Lervåg et al. 2012) and that phonemic awareness instruction produces significant gains in word reading for students with reading difficulties (Ehri et al. 2001; Rice et al. 2022). This predictive relationship holds across the full range of reading ability, not only among students with dyslexia; the phonological deficit hypothesis is therefore fully compatible with the dimensional view adopted in this article, with the severity of phonological weaknesses relating to where students fall along the continuum of word reading difficulty.

Neuroimaging research links these phonological difficulties to atypical function and structure in left‐hemisphere reading networks (Shaywitz et al. 1998). Critically, evidence from children studied before formal reading instruction speaks to questions of cause and effect: pre‐reading children at family risk for dyslexia already show reduced activation in left‐hemisphere posterior regions during phonological processing, before reading experience could have shaped brain development (Ozernov‐Palchik and Gaab 2016; Raschle et al. 2012). Because these differences predate reading instruction and reading failure, they suggest that atypical neural development is associated with the emergence of dyslexia rather than arising solely as a consequence of reduced reading experience, although reading experience itself also shapes the brain.

Family‐risk and longitudinal studies provide convergent evidence. A meta‐analysis established that children at familial risk for dyslexia exhibit phonological deficits well before formal reading instruction begins, with these early difficulties predicting later reading outcomes (Snowling and Melby‐Lervåg 2016). Longitudinal work has similarly shown that preschool phonological skills predict word reading difficulties years later (Van Bergen et al. 2014). Phonologically focused interventions also produce strong effects on word reading (Galuschka et al. 2014; McArthur et al. 2018), further substantiating phonological processing's causal role for most individuals with dyslexia.

Although phonological deficits are present in the large majority of individuals with dyslexia—for example, Saksida et al. (2016) identified a phonological deficit in 97% of a sample of 164 children with dyslexia—they are not always the sole contributor. Multiple‐deficit models propose that dyslexia results from the interaction of several risk factors, with phonological processing being the most common but not the only one (Catts et al. 2024; Pennington 2006). Several of the additional factors implicated, such as rapid automatised naming, oral language and vocabulary, are themselves language‐based. This has led researchers to investigate the broader set of cognitive and linguistic processes that also relate to literacy development.

#### 2.4.2 Broader Cognitive Correlates

Beyond phonological processing, cognitive processes including attention, working memory and processing speed influence literacy development and relate to reading disabilities (e.g., Vellutino et al. 2004). Attention allows learners to focus on relevant stimuli while suppressing distractions (Mateer et al. 2018), as when a student concentrates on a teacher's instruction while ignoring classroom noise. Working memory refers to temporarily storing information while processing something else (Baddeley 2012); it is closely related to attention (Redick et al. 2007), explaining the frequent co‐occurrence of ADHD and working memory deficits (Alderson et al. 2010). For example, decoding a multisyllabic word requires storing each syllable's pronunciation while blending syllables. Processing speed, the time required to receive, process and respond to information, is affected by attention, working memory and phonological awareness (Compton et al. 2001; Schatschneider et al. 2002), and slow processing can produce dysfluent reading by slowing grapheme‐phoneme mapping, blending and word‐meaning retrieval. Many, but not all, students with dyslexia exhibit cognitive processing deficits (Peng et al. 2018; Swanson et al. 2009).

#### 2.4.3 Why Aetiology Matters for Intervention

Understanding the causes of dyslexia is essential for evaluating intervention claims. Because phonological processing deficits are the most well‐established cause, interventions that directly target phonological awareness, letter‐sound correspondences and decoding have the strongest evidence base for remediating dyslexia. Conversely, treatments rooted in unsupported etiological claims, such as visual processing deficits, hemispheric imbalance or generic cognitive malleability, lack evidence for improving reading outcomes. The misalignment between proposed cause and intended remedy is the common thread connecting the unsupported treatments and approaches described in subsequent sections.

## 3 Scientifically Supported Instruction for Students With Dyslexia

The IDA recommends a structured literacy approach that develops word reading and spelling alongside comprehension and written composition. Structured literacy explicitly and systematically teaches the structure of language, from foundational, code‐based skills to higher‐order language comprehension, using methods proven effective for developing the automaticity and fluency needed for proficient reading and writing IDA 2025

Key features of the structured literacy approach include (a) explicit, systematic and sequential teaching of literacy at multiple levels: phonemes, letter‐sound relationships, syllable patterns, decoding, morphemes, vocabulary, sentence structure, paragraph structure and text structure; (b) cumulative practice and ongoing review; (c) a high level of student–teacher interaction; (d) the use of carefully chosen examples and nonexamples; (e) decodable text; and (f) prompt, corrective feedback. (Spear‐Swerling 2018, 202)

Because dyslexia represents the more severe end of a continuum of word reading difficulty rather than a categorically distinct condition, students with dyslexia do not require categorically different instruction than typical readers. All students benefit from structured literacy practices, and most students need this type of instruction to become proficient readers. Students with dyslexia require the same evidence‐based instructional content, but delivered at greater intensity to match the severity of their reading difficulties. Intensification operates along several dimensions, each of which can be calibrated to where a student falls along the continuum of reading difficulty (Fuchs et al. 2017): (a) dosage: increasing the duration and frequency of instructional sessions; (b) group size: reducing student‐to‐teacher ratios to allow for greater individualization and responsive feedback; (c) opportunities to respond and practice: increasing the number of repetitions a student receives within and across lessons to support mastery and retention (Filderman et al. 2018); (d) explicitness: providing more detailed modelling, more carefully sequenced examples and more direct corrective feedback; (e) alignment to specific skill deficits: using diagnostic data to target the precise code‐based and language‐based skills a student has not yet mastered, rather than reteaching the general curriculum; and (f) implementation knowledge and fidelity: delivering instruction by educators with working knowledge of evidence‐based practices and ongoing support to implement those practices with fidelity to their active components. Students at the milder end of the continuum may need only modest intensification along one or two dimensions, while students with more severe and persistent difficulties typically require coordinated intensification across multiple dimensions sustained over a longer period. Table 1 summarises these six dimensions of intensification. The limited use of evidence‐based, structured literacy approaches in U.S. schools (e.g., Hanford 2018; Moats 2014; Piasta et al. 2020; M. S. Seidenberg 2013) and the difficulties teachers face in intensifying interventions (e.g., Datnow and Hubbard 2016; Filderman et al. 2022) may explain, in part, why so many students struggle with decoding and understanding grade‐level texts.

**TABLE 1:** Dimensions of intervention intensification for students with dyslexia.

Dimension	Description	Application for students with dyslexia
Dosage	Duration and frequency of instructional sessions	More minutes per session, more sessions per week and sustained over longer periods than typical classroom instruction
Group size	Student‐to‐teacher ratio during instruction	Smaller groups (1:1 to 1:5) to allow individualization, responsive feedback and sustained engagement
Opportunities to respond and practice	Number of repetitions and practice trials within and across lessons	Increased repetitions of letter‐sound correspondences, decoding patterns and spelling rules to support mastery and retention (Filderman et al. 2018)
Explicitness	Directness of teaching, including modelling, examples and feedback	More detailed teacher modelling, more carefully sequenced examples and nonexamples, and more direct corrective feedback
Alignment to specific skill deficits	Use of diagnostic data to target instruction to a student's specific weaknesses	Diagnostic assessment of phonemic awareness, decoding, fluency, vocabulary and comprehension, with instruction targeted to each student's profile rather than to a fixed scope and sequence
Implementation knowledge and fidelity	Educator knowledge of evidence‐based practices and ability to implement instruction with fidelity to the critical components of each practice	Effective intervention can be delivered by a range of educators—including classroom teachers, special educators, reading specialists, paraeducators and tutors—when they have working knowledge of evidence‐based literacy practices and ongoing support to implement those practices with fidelity to their active components

While the same instructional principles guide effective intervention across developmental stages, the relative emphasis and complexity of each component shift as students mature. In early elementary, students with dyslexia typically require explicit instruction in phoneme blending and segmentation aligned with letter‐sound correspondences, single‐syllable decoding and oral language development. As students' progress, instruction shifts towards multisyllabic decoding, morphology, vocabulary and comprehension of increasingly complex text, while continuing to address unconsolidated foundational skills. Adolescents with severe reading difficulties require continued explicit instruction targeted to their skill profiles, with particular attention to morphology, academic vocabulary and discipline‐specific demands. Table 2 summarises these shifts across early elementary, upper elementary and adolescent stages.

**TABLE 2:** Characteristics of effective literacy intervention for students with Dyslexia across developmental stages.

Component	Early elementary (K–2)	Upper elementary (3–5)	Adolescence (6–12)
Phonics and decoding	Systematic and explicit introduction of alphabet knowledge, letter‐sound correspondences and early phonics skills (e.g., consonant digraphs) blending, and decoding of single‐syllable words; heavy use of decodable text	Systematic and explicit instruction in advanced phonics elements, including vowel‐consonant‐e, vowel digraphs, diphthongs, r‐controlled vowels; repeated practice decoding and encoding single syllable and multisyllabic words in isolation and in connected text; continued use of decodable text when needed for additional practice and repetition	Systematic and explicit instruction in advanced decoding strategies for multisyllabic and morphologically complex words; explicit instruction in syllable types and division patterns; extensive use of complex, authentic texts
Phonemic awareness	Phoneme blending and segmentation in words aligned with explicitly taught letter‐sound correspondences, acknowledging the developmental progression from awareness of the initial sound, final sound and then medial sound.	Phoneme blending and segmentation is practiced by reading and spelling words with more phonemes and multisyllabic words; reading and spelling occur in isolation and connected text; targeted phoneme‐level instruction only for students with persistent deficits	Targeted instruction only for students with persistent deficits; embedded within connected text reading and academic writing experiences, rather than taught in isolation
Morphology	Systematic and explicit instruction in early inflectional morphemes (‐s, ‐ed, ‐ing)	Systematic and explicit instruction in common prefixes, suffixes, and base words to support decoding, spelling, and vocabulary; introduction to common Greek and Latin roots	Continued systematic and explicit instruction in Greek and Latin roots, complex affixes, and morphological problem‐solving for academic vocabulary
Vocabulary	Oral vocabulary instruction emphasising word meaning, supported by read‐alouds and discussion	Systematic and explicit instruction of academic and content‐area vocabulary; word‐learning strategies (context clues, morphemic analysis)	Systematic and explicit instruction of academic and content‐area vocabulary; instructional focus on understanding vocabulary within complex texts and using vocabulary during academic writing
Fluency	Letter‐sound and word‐reading fluency; teacher‐modelled fluent reading; connected text reading practice primarily with decodable texts	Connected text fluency through repeated reading, partner reading, and progress monitoring; gradual transition from decodable to authentic text as students develop sufficient decoding proficiency	Continued fluency support, particularly for students with persistent rate deficits; passage‐level fluency embedded within complex, content‐area reading
Comprehension	Listening comprehension through read‐alouds; oral language development	Reading comprehension strategies (e.g., main idea and summarization); explicit instruction in narrative and expository text structures	Discipline‐specific comprehension strategies; close reading; explicit instruction in argumentation, synthesis, and complex text structures
Spelling and writing	Letter formation, encoding of single‐syllable words aligned with phonics instruction	Encoding of multisyllabic words; sentence‐level writing; integration of spelling with morphology instruction	Advanced spelling patterns; paragraph‐ and essay‐level writing; explicit instruction in syntax, cohesion and discipline‐specific writing conventions

## 4 Treatments and Approaches Not Supported by Scientifically Based Reading Research

In the absence of intervention aligned to scientifically based reading research, individuals with dyslexia are likely to continue experiencing reading difficulties, negatively impacting academic, social/emotional and life outcomes. In the following section, we describe pervasive treatments and approaches not supported by scientifically based reading research, connecting them with their origins in misunderstandings of dyslexia. These practices vary in their relationship to the evidence. Some rest on etiological claims that reading science has directly contradicted, such as visual causes of dyslexia, and have no demonstrated benefit for reading. Others incorporate components that are independently well supported but are distinguished by elements that lack empirical support, or have been studied only in low‐rigour research that does not yet establish their effects. What unites them is that, as commonly promoted and implemented, none has been shown to improve reading outcomes for students with dyslexia beyond what evidence‐based structured literacy instruction provides. Table 3 provides a summary of these practices.

**TABLE 3:** Treatments and approaches not supported by scientifically based reading research: Etiological and treatment claims.

Treatment	Etiological claim	Etiological claim supported?	Treatment effective?
Coloured overlays, tinted lenses and specialised lamps	Visual stress causes dyslexia	No	No
Dyslexic fonts	Letter‐shape processing causes dyslexia	No	No
Vision therapy and ocular exercises	Visual or ocular dysfunction causes dyslexia	No	No
Action video game training	Visual attention deficits cause dyslexia	Contested	No (independent replication failed)
Multisensory instruction (simultaneous VAKT)	Simultaneous activation of visual, auditory and kinesthetic/tactile pathways strengthens reading networks	No	No significant added benefit over non‐multisensory interventions (*g* = 0.20 vs. 0.34, *p* = 0.25; Hall et al. 2023)
Orton‐Gillingham as a packaged approach	The integrated OG package is required for effective intervention	Partial (six of seven defining features have independent support; the multisensory feature does not)	Small, positive, but non‐significant effects on foundational skills (*g* = 0.22, *p* = 0.40) and on vocabulary/comprehension (*g* = 0.14, *p* = 0.59) relative to comparison instruction (Stevens et al. 2021). Effects were interpreted as suggesting promise, not established efficacy; low study quality—including inadequate description of comparison conditions—leaves OG's effectiveness undetermined
Brain training (working memory, processing speed, attention)	Training cognitive processes transfers to reading outcomes	No (near transfer only; no transfer to reading)	No
Whole language and balanced literacy	Reading is acquired naturally through immersion in authentic text	No	Insufficient for students with dyslexia

### 4.1 Vision‐Based Therapies and Interventions

Early dyslexia research focused on whether dyslexia was caused by vision problems or visual perceptual difficulties. Physician Adolph Kussmaul (1877) used the term *word blindness* to describe a patient's inability to read despite normal intelligence (Aaron et al. 2008). Ophthalmologist Rudolph Berlin (1884) coined the term *dyslexia* after observing patients who could not read despite seemingly normal vision; he speculated that brain differences could explain the inability to read, but further explanation eluded him.

In the early 1900s, ophthalmologist James Hinshelwood discussed cases of *congenital word blindness*, claiming the primary disability for these individuals fell in the realm of visual memory for words and letters and described letter reversals as the primary symptom (Hinshelwood 1917). Physician, CJ Thomas, also conducted observations of *congenital word blindness*, recommending one‐on‐one intervention using large wooden letters (Aaron et al. 2008). Finally, Samuel T. Orton (1925, 1937) coined the term *stephosymbolia*, meaning ‘twisted symbol’, to describe difficulties with associating the visual forms of words with spoken forms.

Early research characterising dyslexia as ‘word blindness’, letter/word reversals or ‘twisted symbols’ fueled a misunderstanding that persists today: that dyslexia is a visual disorder causing letters and words to appear backward or float around the page. Current scientifically based reading research provides substantial evidence that dyslexia is not primarily caused by vision problems. The American Academy of Paediatrics (Handler et al. 2011) joint technical report on learning disabilities, dyslexia, and vision concluded that ‘vision problems can interfere with the process of reading, but children with dyslexia or related learning disabilities have the same visual function and ocular health as children without such conditions’ (p. 818). However, claims about visual involvement in dyslexia are not monolithic, and evaluating them requires distinguishing among three related but distinct categories of claim: (a) theories proposing vision or visual attention as a causal mechanism for dyslexia; (b) treatments targeting visual processing as a means of remediating dyslexia; and (c) phenomenological reports of visual or perceptual experiences during reading reported by individuals with dyslexia. Each category requires a different evidentiary evaluation, and conflating them produces persistent misunderstandings.

#### 4.1.1 Visual Causation Theories

Two theories propose visual or visual‐attentional dysfunction as causal mechanisms for dyslexia. The magnocellular deficit theory proposes that dyslexia results from impaired functioning of the magnocellular pathway of the visual system, which processes rapid, low‐contrast and motion‐related visual information (Stein 2018; Stein and Walsh 1997). Proponents argue that magnocellular dysfunction disrupts the fast visual processing required for efficient reading. Some early evidence supported this hypothesis, including histological studies of magnocellular cells in the lateral geniculate nucleus and psychophysical studies reporting reduced contrast sensitivity in dyslexic readers (Livingstone et al. 1991). However, the broader evidence base is inconsistent and contested. A review of contrast sensitivity studies found that results consistent with the theory were outnumbered by studies finding no magnocellular deficit or finding patterns inconsistent with magnocellular dysfunction (Skottun 2000). A meta‐analysis of PET and fMRI activation studies found no evidence for the visual/magnocellular hypothesis when benchmarked against reduced recruitment of brain area V5/MT, the region most associated with magnocellular processing (Paulesu et al. 2014). Olulade et al. (2013) provided particularly compelling evidence that visual magnocellular dysfunction observed in individuals with dyslexia is consequential to impoverished reading experience rather than causal of dyslexia. Although a small group of researchers continues to advance this theory, the preponderance of contemporary evidence does not support magnocellular dysfunction as a primary causal mechanism in dyslexia.

The visual attention span deficit hypothesis proposes that some individuals with dyslexia exhibit difficulty processing the multiple visual elements required for efficient word recognition, independent of phonological deficits (Bosse et al. 2007; Valdois 2022). Proponents argue that this visual‐attentional limitation contributes to dyslexia in a subset of individuals and offers an alternative cognitive account to the phonological deficit hypothesis. The visual attention span hypothesis remains contested, with debate about whether visual attention span tasks measure visual attention specifically or capture other processes such as visual short‐term memory (Valdois 2022). Even if visual attention span contributes to dyslexia in some individuals, this would not displace the phonological deficit as the most well‐established cause; rather, it would be consistent with multiple‐deficit models of dyslexia (Pennington 2006) in which phonological processing is the most common but not the sole contributor.

#### 4.1.2 Vision‐Based and Visual‐Attentional Treatments

The historical misunderstanding of dyslexia as a vision problem gave rise to scientifically unsupported treatments still used today: (a) coloured overlays, lenses or specialised lamps; (b) dyslexic fonts; (c) vision therapy; and more recently, (d) action video game training. Coloured overlays, lenses and lamps purport to reduce visual stress from Meares–Irlen Syndrome or Scotopic Sensitivity (Irlen and Lass 1989; Meares 1980), but individuals with dyslexia are no more susceptible to these conditions than non‐dyslexic individuals (Handler et al. 2011). Suttle et al. (2018) reviewed four systematic reviews of coloured overlays and lenses; three found insufficient evidence of benefit (Albon et al. 2008; Galuschka et al. 2014; Griffiths et al. 2016), while the fourth concluded the approach had promise despite noting poor study quality (Evans et al. 2017).

The American Academy of Paediatrics' joint technical report (Handler et al. 2011) reiterated that vision training, muscle exercises, ocular pursuit and tracking exercises, behavioural/perceptual vision therapy, and training glasses do not directly or indirectly treat dyslexia. Kuster et al. (2018) examined whether students with dyslexia read more accurately and efficiently using a dyslexic font and found that individuals with dyslexia did not read connected text or word lists written in the *Dyslexie* font faster or more accurately than *Arial* or *Times New Roman*. Notably, participants with dyslexia preferred *Arial* and *Times New Roman* over *Dyslexie*, although preference was unrelated to reading performance.

A more recent vision‐based intervention to emerge from the visual attention deficit literature is action video game training. Building on the visual attention span deficit hypothesis, several research teams have proposed that action video games train dynamic visual attention and may produce transfer effects to reading. An early study reported that 12 h of action video game play improved reading speed in Italian children with dyslexia without direct phonological or orthographic instruction (Franceschini et al. 2013). Subsequent studies from the same and related research groups have reported similar effects on reading rate, attentional control and rapid naming (Bertoni et al. 2021; Franceschini et al. 2017; Peters et al. 2021). However, the action video game literature has methodological limitations including small sample sizes and a heavy concentration of studies from a single research network. When an independent research team in Poland tested action video game training against both a phonological video game training condition and a no‐treatment control, they found that all three groups improved comparably, raising the possibility that observed gains reflect general developmental effects, novelty or testing effects rather than a specific treatment effect of action video games (Łuniewska et al. 2018). At present, action video game training does not have a sufficient independent evidence base to be considered a scientifically supported treatment for dyslexia, and its theoretical premise—that visual attention training will produce meaningful transfer to reading—has not been independently replicated outside the research networks that proposed it.

#### 4.1.3 Visual and Perceptual Experiences During Reading

Distinct from theories of visual causation and vision‐based treatments, a body of research documents that some individuals with dyslexia report visual or perceptual experiences during reading, including visual crowding, perceptual instability and reading‐related fatigue (Bertoni et al. 2019; Kristjánsson and Sigurdardottir 2023). These experiences are real and warrant acknowledgment. However, the relationship between these experiences and dyslexia itself remains contested. They may reflect a comorbid perceptual sensitivity—sometimes labelled visual stress, scotopic sensitivity or Meares–Irlen Syndrome—that co‐occurs with dyslexia at elevated rates, a downstream consequence of effortful decoding, or both. Critically, the existence of these phenomenological experiences does not validate causal claims (visual processing as the aetiology of dyslexia) or treatment claims (vision‐based interventions as remediation for dyslexia). The intervention evidence remains the firmer ground: regardless of how these experiences are best explained theoretically, treating them through coloured overlays, tinted lenses, font modifications, vision therapy or attention‐based training does not address the phonological processing deficits underlying dyslexia and does not produce meaningful improvements in reading outcomes (Galuschka et al. 2014; Griffiths et al. 2016; Suttle et al. 2018).

#### 4.1.4 Implications for Practice

Educators, parents and policymakers should be cautious about treatment claims that promise to remediate dyslexia through visual or visual‐attentional means. Although the magnocellular and visual attention span hypotheses have generated substantive research, neither provides a strong empirical basis for replacing or supplementing structured literacy with vision‐ or attention‐based treatments. Instructional time spent on vision‐based interventions is time not spent on the structured literacy instruction students with dyslexia require.

### 4.2 Multisensory Instruction

Early dyslexia research also focused on neurobiological explanations. Samuel T. Orton replaced *stephosymbolia* with *specific reading disability*, which he hypothesised was caused by the failure to establish hemispheric dominance, the functional inequality of the left and right sides of the brain for literacy‐related tasks (Orton 1925, 1937). Influenced by Grace Fernald's kinesthetic work, Orton collaborated with psychologist and teacher Anna Gillingham to develop the Orton–Gillingham (OG) approach for students for whom reading and spelling do not come easily. This approach pioneered direct and explicit letter‐sound and decoding instruction, while heavily emphasising simultaneous use of the visual, auditory, and kinesthetic/tactile (VAKT) activation (Ritchey and Goeke 2006; Stevens et al. 2021).

While systematic and explicit instruction has substantial empirical support, misunderstandings around multisensory instruction persist. For example, Hebb's phrase ‘neurons that fire together wire together’ (Hebb 2005), which describes how repetition strengthens neural pathways, is frequently misquoted to promote simultaneous VAKT activation during instruction for students with dyslexia (e.g., Eyewords Evidence‐Based Multisensory Instruction 2024), rather than its actual meaning of repetition and practice.

Multisensory instruction typically refers to the use of VAKT senses. Despite lacking empirical support, it continues to be regarded by many in the U.S. as a ‘key ingredient’ for teaching students with dyslexia (Stevens et al. 2021). Disagreements may stem from differing definition of multisensory; literacy instruction is innately multisensory if reading involves seeing letters, hearing sounds/words as text is read aloud, and writing to spell (Lane 2022). From this lens, it is difficult to imagine how literacy instruction could be anything other than multisensory.

However, some advocates in the dyslexia community regard multisensory instruction as unique from other literacy interventions, holding the erroneous belief that VAKT senses must be *simultaneously* activated to benefit students with dyslexia (*What is Multisensory Structured Language* 2024). For example, students might concurrently look at a colour‐coordinated phonogram card (visual), produce the corresponding phoneme (auditory), and trace the phonogram on a textured mat while repeating the sound aloud (VAKT). Proponents claim this simultaneous activation strengthens neural pathways and supports attention, retrieval and learning (Birsh 2011; Shams and Seitz 2008), despite the lack of supporting evidence.

Orton–Gillingham (OG) is a commonly used approach incorporating multisensory instruction. OG is a ‘direct, explicit, multisensory, structured, sequential, diagnostic and prescriptive way to teach literacy when reading, writing and spelling do not come easily to individuals, such as those with dyslexia’ (What is the Orton‐Gillingham Approach 2024). The OG approach as a whole has a limited evidence base. An earlier systematic review found the available studies too few and methodologically weak to support OG as evidence‐based (Ritchey and Goeke 2006), and a subsequent meta‐analysis found small, positive, but non‐significant effects on foundational skills (*g* = 0.22) and on vocabulary and comprehension (*g* = 0.14) relative to comparison instruction, interpreting these as suggesting promise rather than established efficacy (Stevens et al. 2021). Despite this weak evidence base, OG remains widely used with students with dyslexia (Uhry and Clark 2005) and is promoted and mandated in U.S. state dyslexia legislation (e.g., Davis Dyslexia Association International 2024).

It is important to distinguish between the individual features that comprise the OG approach. Six of the seven defining features—*direct*, *explicit*, *structured*, *sequential*, *diagnostic* and *prescriptive* instruction—have strong, independent empirical support for improving reading outcomes of students with or at risk for dyslexia (Austin et al. 2023). These six features overlap substantially with other structured literacy interventions, which are supported by scientifically based reading research. The seventh feature—*multisensory* instruction, defined as the simultaneous activation of visual, auditory and kinesthetic/tactile senses—is the feature that lacks independent empirical support and that distinguishes OG from other structured literacy approaches. This claim has since been examined directly. In a meta‐analysis of reading interventions for elementary students with or at risk for dyslexia, interventions explicitly described as multisensory did not produce significantly larger effects than interventions not so described (*g* = 0.20 vs. 0.34, *p* = 0.25); the authors concluded there is insufficient evidence to justify requiring multisensory programmes in place of other evidence‐based, structured approaches, while noting this comparison was underpowered and warrants further study (Hall et al. 2023). Further studies designed to isolate the contribution of multisensory instruction from the other instructional features are needed to confirm whether multisensory activation provides any benefit over and above the contributions of direct, explicit, structured, sequential, diagnostic and prescriptive instruction.

In short, OG appears to produce reading gains primarily because it incorporates the structured literacy features that are independently supported by research, not because of its multisensory component. When OG is used with students with dyslexia, the active ingredients are likely the structured literacy features OG shares with other evidence‐based approaches rather than the multisensory feature that is unique to OG.

Beyond OG, multisensory instruction has come to represent a wide array of practices, including spelling using a variety of mediums (sky writing, sand, rice, shaving cream, sandpaper and textured mats), finger tapping to segment words into phonemes, and clapping to segment syllables. Some of these practices closely align with research‐based instruction. For example, integrating spelling instruction within reading instruction has wide empirical support (Graham and Santangelo 2014; Graham et al. 2017), though the benefit likely reflects shared knowledge and cognitive processes (Clemens and Vaughn 2023) rather than special mediums or *simultaneous* VAKT activation. Similarly, finger tapping to segment phonemes draws on phonemic awareness, which is strongly associated with skilled reading and improved by phonemic awareness instruction (Ehri et al. 2001; Erbeli et al. 2018; Melby‐Lervåg et al. 2012; Rice et al. 2022); the benefit is likely due to the phonemic awareness focus, not the finger tapping or simultaneous VAKT activation.

Other multisensory practices lack close alignment with research‐based instruction. Writing in the sky, on sandpaper, or on a textured mat leaves no written trace for teachers to evaluate spelling accuracy and provide corrective feedback, and spelling in sand, rice or shaving cream leaves a trace but does not require the pencil grip, pressure or motor control of standard writing. No experimental research demonstrates that these practices improve reading outcomes for students with dyslexia, and educators must consider the opportunity cost of instructional time spent on activities lacking empirical support.

### 4.3 Interventions Targeting Other Cognitive Skills (Or Risk Factors)

Given the close relationship between cognitive processing and literacy achievement, brain training programmes emerged purporting to remediate cognitive processing deficits for individuals with dyslexia. Programmes target the same cognitive processes implicated in literacy difficulties: working memory, processing speed and attention. The cumulative evidence across all three domains, however, points to a consistent pattern: cognitive training produces near transfer to tasks resembling the training itself, but far transfer to untrained cognitive abilities or to academic outcomes such as reading is rare or absent.

Working memory training has been the most extensively studied form of cognitive training. Two early systematic reviews concluded working memory training can produce short‐term benefits on working memory tasks similar to the training but have no significant effects on untrained working memory tasks, long‐term working memory or reading (Hulme and Melby‐Lervåg 2012; Shipstead et al. 2012). A subsequent meta‐analysis of 50 studies of Cogmed working memory training (637 effect sizes) reached the same conclusion, finding small to medium effects on memory tasks similar to the training (near transfer) but near‐zero effects on attention, intelligence, language ability and academic achievement (Aksayli et al. 2019). Findings have been replicated in studies including participants with or at risk for dyslexia (e.g., Maehler et al. 2019; Peng and Fuchs 2016).

Processing speed training shows a similar pattern. Although deficits in processing speed are commonly observed in students with dyslexia and have been hypothesised to contribute to slow and dysfluent reading, training programmes targeting processing speed have not produced reliable transfer to reading outcomes for students with dyslexia. Where reading improvements have been reported following processing speed training, they typically occur in studies that combined cognitive training with reading instruction, making it impossible to isolate the unique contribution of the cognitive training itself (Cancer et al. 2020).

Attention training programmes have likewise failed to demonstrate far transfer to reading. A meta‐analysis of cognitive training in children with ADHD found that working memory training programmes produced near transfer to working memory tasks but no transfer to attention, inhibitory control or to academic outcomes including reading (Cortese et al. 2015). Studies that have specifically examined attention training in typically developing children and children with reading difficulties have found similar results, with no significant effect of attention training on phonological or reading skills (Loosli et al. 2012; Moreau et al. 2016).

In summary, despite a strong theoretical rationale linking cognitive processes deficits to reading difficulties, brain training programmes targeting working memory, processing speed or attention have not been shown to produce meaningful improvements in reading outcomes for students with or at risk for dyslexia. The pattern of near transfer without far transfer is consistent across cognitive domains and across populations. This pattern also bears on causation: if strengthening these cognitive skills through training does not improve reading, the association between such skills and reading difficulty is unlikely to be straightforwardly causal. The observed cognitive weaknesses may instead be correlates of, or consequences of, reduced reading experience rather than remediable causes of dyslexia. Instructional time spent on brain training programmes is instructional time not spent on the structured literacy instruction students with dyslexia require to develop proficient reading.

### 4.4 Whole Language and Balanced Literacy Interventions

The *whole language* approach originated in the 1800s with Horace Mann, who cautioned against teaching children to sound out words for fear of detracting from meaning (Harris 1896). The approach immerses students in reading and writing under the assumption that children will acquire necessary phonics skills through authentic reading. By the 1950s, whole language was the primary U.S. method for teaching reading, encouraging sight word memorization and use of context and picture cues, and producing the *Dick and Jane* ‘look–say’ readers (Hiebert 2011). Goodman (1967) characterised reading as a ‘psycholinguistic guessing game’, rejecting reading as precise letter‐by‐letter identification and instead encouraging context‐based reading for meaning. Despite research showing that poor readers, not strong readers, rely on whole language strategies (Chall 1967, 1983), the approach continued, rebranded as *balanced literacy*.

Fountas and Pinnell (2012–2013) describe *balanced literacy* as a philosophical orientation combining code‐focused and meaning‐focused instruction. The approach has proliferated with varied interpretations, all rooted in the belief that proficient readers do not need to use sublexical orthography and phonology to ‘sound out’ words. Consequently, proponents presume early readers do not require systematic, explicit phonics instruction (Fletcher et al. 2021). Balanced literacy, instead, immerses children in age‐appropriate literature, teaches sight word recognition and emphasises meaning. When phonics is taught, it is typically provided minimally and incidentally, rather than systematically (Fountas and Pinnell 2012–2013).

For example, during systematic and explicit phonics instruction within structured literacy, a teacher introduces a letter combination and teaches the associated phoneme (‘The letters *ow* can make the /ō/ sound’), models decoding (‘/s/ /n/ /ō/ blends to *snow*’), provides guided practice with words such as *bow*, *blow*, *crow*, and *grow*, and gives independent practice reading words and decodable text containing the targeted pattern.

In contrast, during whole language or balanced literacy instruction, a student may encounter an unfamiliar word while reading: ‘The clown has a big, red *bow*’. If the student misreads *bow* using the /ow/ sound from *clown*, the teacher prompts the student to think about whether the sentence makes sense or to check the picture for help. If prompts produce the correct pronunciation, reading continues; if not, the teacher briefly explains the alternative pronunciation. This type of instruction does not provide the repetitions many students with dyslexia require for mastery, and it focuses on meaning rather than sublexical skills like letter‐sound correspondences. Because students with limited decoding cannot read many words in authentic texts, balanced literacy often uses predictable texts that support word reading through repetition and pictures (e.g., ‘I see apples at the market. I see bananas at the market’), encouraging guessing from text structure, context and pictures rather than decoding.

Despite continued reading wars, research is clear. Systematic reviews of scientifically based reading research demonstrate that systematic and explicit phonics instruction yields moderate, significant gains in foundational reading skills and comprehension for students with reading difficulties and disabilities (Ehri et al. 2001; Galuschka et al. 2014; McArthur et al. 2018). According to Stanovich (2000), research has conclusively proven that direct instruction in the alphabetic principle facilitates the acquisition of skilled reading.

Proponents of explicit phonics also recognize that skilled reading requires instruction beyond phonics, despite misattributions equating the science of reading with phonics alone. Morris et al. (2012) randomly assigned Grade 2–3 students with significant word reading difficulties to four conditions: traditional explicit phonics, explicit phonics with additional strategies (reading by analogy, peeling affixes, identifying familiar word parts and using variable vowel pronunciations), explicit phonics alongside morphology and vocabulary instruction or a math‐instruction comparison. The largest effects were observed for approaches integrating more comprehensive word study within explicit phonics. Subsequent research demonstrates that integrating word meaning instruction with word reading significantly improves word reading accuracy, efficiency, and word meaning knowledge for students with dyslexia in Grades 4–5 (Austin et al. 2022).

Discussion must move beyond debates between explicit phonics and whole language or balanced literacy. Research is firmly settled that all students benefit from systematic and explicit phonics instruction, many require this type of instruction to become proficient readers, and students with dyslexia often require intensive interventions in explicit phonics with many repetitions. Students with dyslexia require structured literacy practices that include explicit phonics instruction and the use of decodable text, but structured literacy also includes direct and explicit instruction across other forms of sublexical instruction and other aspects of language and knowledge. Future research must focus on integrating evidence‐based practices into comprehensive reading interventions individualised to students' needs (Fletcher et al. 2021).

## 5 Factors Influencing Identifying Scientifically Supported Curricula

Multiple factors complicate efforts to ensure scientifically based reading research informs instruction. Inaccurate information falls into two categories: misinformation (inaccurate information spread without intent to mislead) and disinformation (deliberately misleading information spread with intent to deceive; Vraga and Bode 2020; Wardle and Derakhshan 2017). The proliferation of both hinders implementation of research‐based interventions, making it essential to examine the sources and mechanisms of mis/disinformation.

Misinformation often originates from well‐intentioned individuals lacking knowledge to guide teachers towards research‐based practices (Kendeou and Johnson 2024). *Faux experts* have a shallow understanding of reading research and may inadvertently share information without understanding rigorous peer‐review processes or extend research claims beyond actual findings. *Programme disciples and enthusiasts* are committed to a particular programme or approach lacking scientific support; for example, a parent observing a tutor use a structured literacy intervention that includes multisensory practices may incorrectly attribute a child's progress to the multisensory features and promote them as essential for children with dyslexia.

Disinformation, in contrast, involves deliberate intent to mislead, often for monetary profit (Wardle and Derakhshan 2017). *Educelebrities* with large social media followings may share information motivated by likes, audience reach, and sales rather than scientifically based reading research, while *snake‐oil salesmen* peddle curricula, programmes, and training services lacking scientific support for personal gain. Many widely used curricula and even publishing companies' offerings lack empirical support (Hanford 2020; Hanford and Peak 2022; Peak 2022; Stevens et al. 2021). The use of scientifically unsupported treatments carries real costs in money, time and lost opportunity to deliver intervention supported by research.

Several interconnected mechanisms drive the spread of mis/disinformation. Social media algorithms feed users more of the dyslexia content they already engage with, regardless of scientific evidence (Rodrigues et al. 2024), creating echo chambers within groups holding similar beliefs. Humans are also susceptible to confirmation bias, the tendency to favour information confirming existing beliefs (Mynatt et al. 1977; Wason 1960). Within echo chambers, the volume of similar information is easily misinterpreted as evidence that a programme is research‐supported, and groupthink encourages *programme disciples and enthusiasts* to accept shared claims as fact without questioning the evidence. The confluence of algorithmic amplification, confirmation bias and groupthink makes it difficult for individuals without substantial knowledge to distinguish evidence‐based practices from unsubstantiated claims.

Research supports both proactive and reactive strategies for combating mis/disinformation. Proactive strategies—cultivating critical thinking in educators and parents, implementing resource verification protocols and prebunking (forewarning stakeholders about common dyslexia misinformation and providing research‐based counterarguments)—have proven effective across fields (Boman 2023; Compton 2024) and can prevent the adoption of ineffective interventions before they gain traction. Reactive measures, such as debunking after misinformation has spread and encouraging open dialogue about evidence (Jin et al. 2020; Van der Meer and Jin 2020), are also valuable, but proactive approaches better prevent dissemination of non‐evidence‐based practices in the first place.

Conducting rigorous evaluation of widely used curricula presents real challenges. The vast number of programmes used in schools, frequently revised into new editions, would require extensive time and funding to evaluate. The U.S. What Works Clearinghouse (WWC) reviews programmes that have been widely studied, but many programmes commonly used in schools remain unreviewed because the WWC must prioritize interventions with a large existing research base. Companies likely to profit from positive findings often fund research studies, introducing conflict‐of‐interest concerns. Regardless of cause, the continued use of unsupported curricula represents a loss of educational opportunity for students with dyslexia.

## 6 Concluding Thoughts

The scientific study of dyslexia has produced substantial consensus, even as important questions remain. Current definitions converge in describing dyslexia as a difficulty with accurate and fluent word reading and spelling, not attributable to intellectual disability or inadequate instruction, that varies along a continuum of severity. Phonological processing deficits are the most well‐established contributor, present in the large majority of individuals with dyslexia, with additional cognitive and language‐based factors implicated in multiple‐deficit accounts. Evidence from children studied before formal reading instruction indicates that associated neurodevelopmental differences are present before reading experience could have shaped them, suggesting they are associated with the emergence of dyslexia rather than arising solely as its consequence. What remains less settled is agreement on aetiology and on the boundaries of the construct—but these debates concern how best to explain and demarcate dyslexia, not whether reading difficulty is real or remediable.

The evidence is likewise clear about intervention. Students with dyslexia benefit from the same evidence‐based, structured literacy instruction that supports reading development broadly, delivered with greater intensity matched to the severity of their difficulties. In contrast, the treatments and approaches examined here are not supported by scientifically based reading research. Some rest on etiological claims that reading science has contradicted: coloured overlays and tinted lenses, dyslexic fonts, and vision therapy assume a visual basis for dyslexia, and action video game training and cognitive or ‘brain’ training programmes assume that training visual attention or general cognitive processes will transfer to reading—claims for which the evidence is absent or, in the case of independent replication, unsupportive. Other approaches to instruction share research‐based components but are distinguished by elements that lack empirical support: multisensory instruction and the Orton–Gillingham approach incorporate explicit, systematic instruction that is well supported, yet the multisensory activation that distinguishes them has not been shown to add benefit, and whole language and balanced literacy approaches lack the systematic, explicit foundational‐skills instruction that reading science supports. As commonly promoted and implemented, none of these treatments or approaches has been shown to improve reading outcomes beyond what structured literacy instruction provides, and time spent on them is time not spent on the instruction students with dyslexia require.

U.S. education stakeholders, including state and local education agencies, schools, administrators, teachers and parents of children with dyslexia, must be critical consumers of educational information (Filderman et al. 2023). Not all readily available information is accurate or applicable to a heterogeneous population differing in symptom severity, cognitive processing, language backgrounds and achievement profiles. In today's digital landscape of widespread mis/disinformation, advocates can take proactive and reactive steps to protect others from deception and identify trustworthy sources, but all education stakeholders must promote policies and practices grounded in scientifically based reading research.

## Funding

The authors have nothing to report.

## Ethics Statement

The authors have nothing to report.

## Consent

The authors have nothing to report.

## Conflicts of Interest

The authors declare no conflicts of interest.

## Data Availability

Data sharing not applicable to this article as no datasets were generated or analysed during the current study.
